# O.5.1-8 Modal shift towards the e-bike: preliminary results from a systematic review on factors influencing the use of e-bikes

**DOI:** 10.1093/eurpub/ckad133.238

**Published:** 2023-09-11

**Authors:** Eva Christina Lorenz, Buse Yürür, Laura Bieniosek, Peter Gelius, Karim Abu-Omar

**Affiliations:** Friedrich-Alexander University Erlangen-Nürnberg, Germany; eva.lorenz@fau.de; Friedrich-Alexander University Erlangen-Nürnberg, Germany; eva.lorenz@fau.de; Friedrich-Alexander University Erlangen-Nürnberg, Germany; eva.lorenz@fau.de; Friedrich-Alexander University Erlangen-Nürnberg, Germany; eva.lorenz@fau.de; Friedrich-Alexander University Erlangen-Nürnberg, Germany; eva.lorenz@fau.de

## Abstract

**Background:**

In the last decade, the use of electric bikes (e-bikes) has become increasingly common due to rapid advances in technology. This has led to an increased number of publications aimed at investigating individual factors and beliefs that influence a modal shift towards e-bikes. To our knowledge, there is currently no review summarizing the evidence on individual motivations, facilitators, and barriers for e-bike use. This review aims at providing an overview of the current state of the art regarding individual choices and intrinsic factors to change from other modes of transport to e-bikes.

**Methods:**

Searching for the term e-bike its synonyms and permutations on the databases SCOPUS, PubMed, Web of Science, and PsychINFO resulted in a total of 3,317 hits after removing duplicates. Screening of title/abstract and full-text screening is currently being conducted independently by three researchers using Covidence. Studies reporting on personal decision-making factors and reasons for a modal shift to a private e-bike are considered eligible for inclusion in this study. Studies focusing on public e-bike services are excluded. Studies will be analyzed regarding (a) research quality, (b) factors and reasons for model shift, and (c) information about the investigated populations.

**Results:**

Preliminary results show a substantial increase in scientific studies related to e-bikes. After the title and abstract screening, 121 studies were included for full-text screening. Preliminary results allow assumptions about two different aspects: (a) Most studies connected the modal shift to e-bikes to facilitators rather than to barriers. Financial, temporal and social factors, along with enjoyment, health and ease of commuting seem to be among the motivators to change to an e-bike as a means of transportation, (b) the majority of studies focused on middle-aged and older populations.

**Conclusion:**

The identified articles and their summary show the increased relevance of e-bikes in current research and public health. Our results may contribute to understanding the subjective reasons why people change their mode of transport to the e-bike. This knowledge may be useful for implementing public health interventions, infrastructure developments, and policies. More research is needed to better understand these factors and their impact.

